# Wheel-running activity modulates circadian organization and the daily rhythm of eating behavior

**DOI:** 10.3389/fpsyg.2014.00177

**Published:** 2014-03-04

**Authors:** Julie S. Pendergast, Katrina L. Branecky, Roya Huang, Kevin D. Niswender, Shin Yamazaki

**Affiliations:** ^1^Department of Biological Sciences, Vanderbilt UniversityNashville, TN, USA; ^2^Gunn High SchoolPalo Alto, CA, USA; ^3^3VA Tennessee Valley Healthcare SystemNashville, TN, USA; ^4^Division of Diabetes, Endocrinology and Metabolism, Department of Medicine, Vanderbilt University School of MedicineNashville, TN, USA

**Keywords:** circadian, C57BL/6J, mice, voluntary exercise, eating behavior, metabolism, obesity, liver

## Abstract

Consumption of high-fat diet acutely alters the daily rhythm of eating behavior and circadian organization (the phase relationship between oscillators in central and peripheral tissues) in mice. Voluntary wheel-running activity counteracts the obesogenic effects of high-fat diet and also modulates circadian rhythms in mice. In this study, we sought to determine whether voluntary wheel-running activity could prevent the proximate effects of high-fat diet consumption on circadian organization and behavioral rhythms in mice. Mice were housed with locked or freely rotating running wheels and fed chow or high-fat diet for 1 week and rhythms of locomotor activity, eating behavior, and molecular timekeeping (PERIOD2::LUCIFERASE luminescence rhythms) in *ex vivo* tissues were measured. Wheel-running activity delayed the phase of the liver rhythm by 4 h in both chow- and high-fat diet-fed mice. The delayed liver phase was specific to wheel-running activity since an enriched environment without the running wheel did not alter the phase of the liver rhythm. In addition, wheel-running activity modulated the effect of high-fat diet consumption on the daily rhythm of eating behavior. While high-fat diet consumption caused eating events to be more evenly dispersed across the 24 h-day in both locked-wheel and wheel-running mice, the effect of high-fat diet was much less pronounced in wheel-running mice. Together these data demonstrate that wheel-running activity is a salient factor that modulates liver phase and eating behavior rhythms in both chow- and high-fat-diet fed mice. Wheel-running activity in mice is both a source of exercise and a self-motivating, rewarding behavior. Understanding the putative reward-related mechanisms whereby wheel-running activity alters circadian rhythms could have implications for human obesity since palatable food and exercise may modulate similar reward circuits.

## Introduction

Circadian rhythms of physiology and behavior have endogenous ~24-h periods that synchronize to environmental cycles of light/dark and food availability (Takahashi et al., 2001). In mammals, the master circadian clock, located in the suprachiasmatic nucleus (SCN) of the anterior hypothalamus, receives direct input from the retina about the environmental light-dark cycle and coordinates the timing of clocks in extra-SCN brain regions and peripheral tissues (Yamazaki et al., 2000; Abe et al., 2002; Yoo et al., 2004). Changes in environmental cycles of light/dark and timing of food intake, such as occurs with transmeridian air travel or rotating shift-work, induces internal desynchronization of circadian clocks so that physiological and behavioral rhythms are no longer optimally tuned to environmental conditions (Yamazaki et al., 2000).

Mice fed high-fat diet develop diet-induced obesity, which is characterized by increased body weight and fat mass (Surwit et al., 1988; Krawczewski Carhuatanta et al., 2011). Chronic high-fat diet consumption also alters daily rhythms of locomotor activity, body temperature, and food intake in mice (Kohsaka et al., 2007; Mendoza et al., 2008). We recently investigated the proximate effects of high-fat diet consumption on daily rhythms of behavior and on the phase relationship between tissue oscillators in mice (i.e., circadian organization). We measured the phases of the circadian gene fusion protein reporter rhythms [PERIOD2::LUCIFERASE (PER2::LUC)] in explanted tissues and found that most tissue clocks, including the SCN, arcuate nucleus of the hypothalamus, and white adipose tissue, are resistant to the acute effects of high-fat diet consumption (Pendergast et al., 2013). In contrast, the phase of the liver rhythm is markedly advanced after only 1 week of high-fat diet consumption. The expression of transcripts and metabolites in the liver are profoundly altered after only 3 days of high-fat diet consumption, demonstrating the acute metabolic consequences of high-fat diet on the mouse liver (Eckel-Mahan et al., 2013). In addition to the alteration of the liver rhythm, the daily rhythm of eating behavior is immediately altered by high-fat diet as eating events become uniformly distributed across the day and night.

Voluntary wheel-running activity decreases fat mass and increases energy expenditure in mice fed high-fat diets (Bell et al., 1995; Krawczewski Carhuatanta et al., 2011; Scarpace et al., 2012; Meek et al., 2013). Wheel-running activity also alters circadian rhythms in mice. For example, wheel-running activity shortens the period of the activity rhythm and accelerates re-entrainment to shifted light-dark cycles (Harrington et al., 2007; Leise et al., 2013). Wheel-running activity also alters the phase of the liver rhythm in chow-fed mice (Schroeder et al., 2012). In this study, we sought to determine whether voluntary wheel-running activity counteracts the proximate effects of high-fat diet consumption on circadian organization and behavioral rhythms in mice.

## Materials and methods

### Animals

Male C57BL/6J heterozygous PER2::LUCIFERASE mice (Yoo et al., 2004) (N21–23; backcrossed to C57BL/6J mice from The Jackson Laboratory) were bred in the Vanderbilt University animal facility in a 12 h-light/12 h-dark cycle (12L:12D; light intensity ~350 lux). Breeders were fed chow (13.5% kcal from fat, LabDiet 5L0D) and water *ad libitum*. Genotyping was performed by measuring light emission from a tail piece with a luminometer. Mice were euthanized by cervical dislocation followed by decapitation. The Vanderbilt University Institutional Animal Care and Use Committee approved all experiments (M/08/096).

### Experimental protocol

At 21 days old, male heterozygous PER2::LUC mice were weaned and group housed (2–4 mice per cage). Chow and water were provided *ad libitum*. At 7 weeks old, mice were singly housed in cages (33 × 17 × 14 cm) and provided with chow *ad libitum*. The cages contained either locked wheels (wheels could not rotate) or freely rotating wheels and were housed in light-tight boxes in 12L:12D (light intensity 200–300 lux; temperature inside light-tight boxes: 25.5 ± 1.5°C). At 8 weeks old, the chow was replaced with either high-fat diet (45% kcal from fat; Research Diets D01060502) or with fresh chow. Food was changed within 3 h of lights off. One mouse was excluded from the analysis because it did not have any eating events for 12 h after high-fat diet was placed in the food hopper. To evaluate the effect of environmental enrichment without a running wheel, the experiment was performed as described above except that mice were single-housed in empty cages or in cages with 1 paper-based refuge hut and 2 sheets of nesting paper. At 9 weeks old, PER2::LUC expression was measured in *ex vivo* tissues. Body weight and food were measured at 7, 8, and 9 weeks old within 3 h before lights off. Total kcal consumed was calculated based on the grams of food consumed (chow: 3.02 kcal/g metabolizable energy; 45% HFD: 4.73 kcal/g).

### Luminescence recording

Cultures were prepared within 1.5 h before lights off as we have previously described (Yamazaki and Takahashi, 2005; Pendergast et al., 2013), “The gonadal white adipose tissue (surrounding the gonads; WAT), liver, lung, spleen, aorta, pituitary, SCN, arcuate complex (containing the arcuate nucleus of the hypothalamus and ependymal cell layer as described previously Guilding et al., 2009) were collected from the same mouse.” We chose tissues that regulate metabolism (liver, WAT, arcuate), food intake (arcuate), cardiovascular function (aorta, lung), immune responses (spleen), hormone secretion (pituitary), and the master circadian pacemaker (SCN). As we previously described (Pendergast et al., 2013), “Bioluminescence was monitored in real-time (at 36.8 ± 0.02°C) with the LumiCycle (Actimetrics), and photon counts were integrated over 10-min intervals. LumiCycle software (Actimetrics) was used to subtract the 24-h moving average from the raw luminescence data and to smooth the data by 0.5-h adjacent averaging. To determine period and phase, the detrended and smoothed data were exported to ClockLab (Actimetrics). The period was determined by fitting a regression line to the acrophase of at least 3 days of the PER2::LUC rhythm. The period of the rhythm in liver explants spontaneously changes after 3–4 cycles in culture. The periods reported for liver (Table 1) are the first periods measured in culture. The phase was determined from the peak of PER2::LUC expression during the interval between 12 and 36 h in culture. LumiCycle software was used to determine the amplitude of the same cycle used to determine phase for each tissue using the sine fit curve-fitting method. Only one cycle was analyzed for amplitude because the period and damping rate of PER2::LUC expression in liver are not constant (Pendergast and Yamazaki, 2012). Samples with a goodness of fit less than 90% were excluded (the resulting number of samples analyzed for amplitude are reported in Table 1). The amplitudes of the rhythms from arcuate explants could not be analyzed because the goodness of fit was always less than 90% because the bioluminescence traces were noisy due to the low level of light emission from the tissue.”

**Table 1:** **Circadian parameters of bioluminescence rhythms in *ex vivo* tissues**.

		**Locked wheel**		**Free wheel**		
	**Tissue**	**Chow: mean ± *SD* (*n*)**	**HFD: mean ± *SD* (*n*)**	**Chow: mean ± *SD* (*n*)**	**HFD:**	P^*^
**Period (h)**	SCN	24.34±0.25 (7)	24.35±0.11 (7)	24.29±0.24 (6)	24.23±0.19 (6)	NS
	Pituitary	23.37±0.27 (6)	23.73±0.59 (7)	23.47±0.47 (6)	23.46±0.50 (6)	NS
	Liver	21.59±0.66 (5)	21.28 h±0.60 (5)	20.68±1.03 (5)	20.24±0.54 (6)	Wheel: *F*_(1, 17)_ = 9.52 *p* < 0.01
	Lung	23.98±0.35 (6)	23.99±0.45 (7)	24.00±0.40 (6)	23.76±0.18 (6)	NS
	Spleen	24.40±0.44 (6)	24.34±0.33 (7)	24.42±0.83 (6)	24.15±0.34 (6)	NS
	Aorta	24.54±0.17 (4)	24.53±0.33 (3)	24.03±0.36 (6)	23.91±0.49 (6)	Wheel: *F*_(1, 15)_ = 9.66 *p* < 0.01
	Arcuate complex	23.37±0.88 (3)	23.17±0.51 (5)	22.53±0.36 (4)	22.35±0.67 (4)	Wheel: *F*_(1, 12)_ = 7.40 *p* = 0.01
	Gonadal WAT	25.85±0.54 (4)	25.59±0.77 (6)	25.62±0.51 (6)	25.74±0.43 (6)	NS
**Amplitude of bioluminescence (counts/s)**	SCN	36.03±14.35 (7)	27.61±11.79 (7)	48.89±12.90 (6)	52.17±33.84 (6)	Wheel: *F*_(1, 22)_ = 5.77 *p* = 0.03
	Pituitary	37.61±11.16 (7)	29.15±11.23 (6)	54.58±26.04 (6)	37.91±10.24 (6)	NS
	Liver	9.43±5.38 (6)	18.32±13.82 (6)	8.97±4.29 (5)	10.96±10.62 (6)	NS
	Lung	26.71±8.31 (7)	21.89±8.68 (7)	34.47±12.34 (6)	22.15±8.69 (6)	Diet: *F*_(1, 22)_ = 5.21 *p* = 0.03
	Spleen	23.58±9.98 (7)	21.57±6.23 (7)	30.14±12.92 (6)	27.24±5.85 (6)	NS
	Aorta	25.61±13.18 (3)	15.70±2.59 (3)	38.32±15.74 (5)	34.15±19.65 (6)	NS
	Arcuate complex	ND	ND	ND	ND	ND
	Gonadal WAT	11.27±6.3 (6)	15.81±7.03 (3)	15.04±6.33 (6)	9.13±9.39 (6)	NS

* *The data were compared by 2 × 2 factorial analysis. None of the tissues had a significant interaction between wheel and diet. Some tissues showed a significant effect of wheel or diet, as reported in the table. NS indicates that no significant differences were found*.

### Locomotor activity and eating behavior

We measured general activity and eating behavior according to our previously reported method (Pendergast et al., 2013). General activity was monitored every minute with passive infrared sensors (sensors record a maximum of 1 count every 6 s; model 007.1, Visonic LTD) and the number of wheel revolutions was recorded every minute (Figure S1) using Clocklab (Actimetrics). One mouse was excluded from the analysis because it ran fewer than 10,000 revolutions/day on Day 7 of the protocol. As we previously described (Pendergast et al., 2013), “An infrared video camera (PYLE PLCM22IR Flush Mount Rear View Camera with 0.5 Lux Night Vision, Pyle Audio Inc, Brooklyn, NY, USA) was connected to a computer with the VideoSecu 4 Channel PCI DVR Card for CCTV Home Security Surveillance System C53 (VideoSecu, Carrollton, TX). DVR software by EYEONET (AMETA International Co. Ltd, Markham, Ontario, Canada) was used to record and analyze the images. To reduce the size of the video file, images were recorded only when the mouse approached the feeder. When the mouse entered a defined region around the feeder, images were captured 1 frame every second for 5 s. Observers (Julie S. Pendergast, Katrina L. Branecky) watched the videos and coded eating behavior in 1-min bins. Eating behavior was defined based on the following criteria: The mouse either: (1) took food from the feeder with its mouth or hands; or (2) moved food in the feeder with its mouth; or (3) ate food in its paws away from the feeder; and this eating behavior persisted for 3 or more seconds (to distinguish eating from other behaviors such as sniffing). When eating behavior occurred, that 1-min was coded as “1” (a 1-min bin could only have a maximum value of “1” regardless of whether eating persisted for 3 s or 1 min), while 1-min bins without eating behavior were coded as “0.” General activity and wheel-running activity were plotted similarly to eating behavior, such that each 1-min bin was coded as either “1” when activity occurred or “0” when there was no activity” (Pendergast et al., 2013). For each mouse, eating, general activity, and wheel-running events during 1 day of chow (Day 7) and during 1 day of high-fat diet (Day 9) consumption were plotted in circular histogram plots in 2.5° bins (10-min) using Oriana 4.0 software (Kovach Computing Services, Wales, UK). Grand mean vectors were calculated for each group (*n* = 5 mice/group).

### Statistical analysis

A 2 × 2 factorial treatment arrangement followed by *post-hoc* Fisher's least significant difference (LSD) tests were used to analyze body weight gain, food intake, and the phases, periods, and amplitudes of tissue PER2::LUC rhythms using SPSS 13.0. Independent *t*-tests (two-tailed) were used to compare body weight, food intake, and the phases of liver PER2::LUC rhythms for the environmental enrichment experiment. Circular data were analyzed and plotted using Oriana 4.0. Rayleigh's Uniformity test was used to determine if the activity, wheel-running, and eating events of individual mice had a significant non-uniform direction (for individual mice). Hotelling's one sample test was used to test if there was a significant mean direction (for grand mean vectors). The mean angle (μ) ± circular standard deviation *(SD)* and vector length (r; degree of clustering) are reported. The grand mean vectors could not be compared by circular Two-Way ANOVA because they violated the assumptions of the test (the concentration, k, was not equivalent between groups and κ was not ≥ 2). All data are the mean ± *SD*. Significance was ascribed at *p* < 0.05.

## Results

### Effects of high-fat diet and wheel-running activity on weight gain and food intake

**Figure 1:**
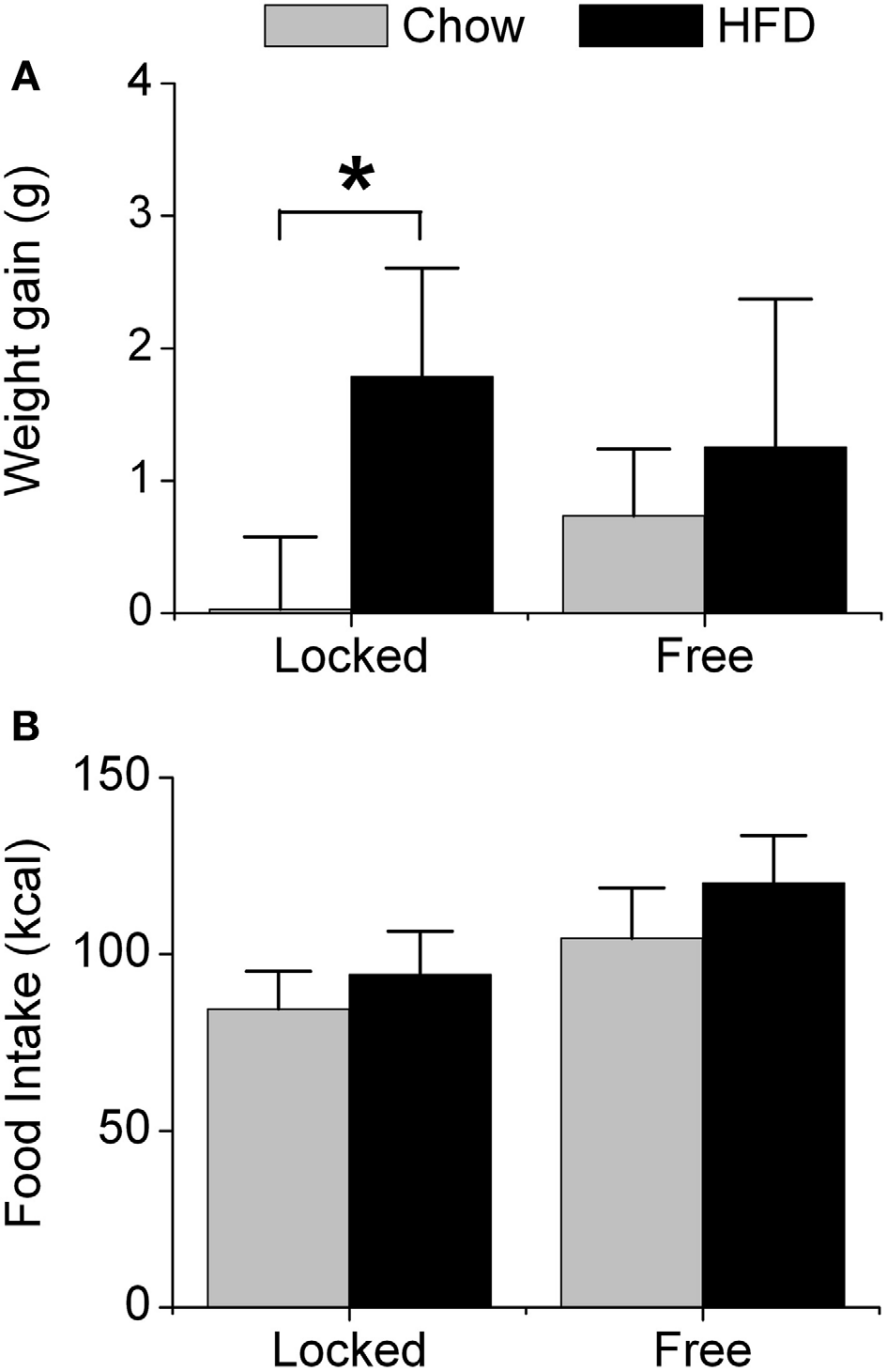
**Acute effect of wheel-running activity on body weight and food intake**. Male heterozygous PER2::LUC mice were single-housed at 7 weeks old in cages with either locked or freely rotating running wheels and provided with chow *ad libitum* for 1 week. At 8 weeks old, mice were provided with fresh chow (gray) or high-fat diet (HFD; black) for 1 week. The mean (±*SD*) weight gain (**A**; grams) and food intake (**B**; kcal metabolizable energy) were determined during 1 week of chow or HFD feeding. There was a main effect of diet [*F*_(1, 32)_ = 14.93, *p* < 0.001] and a significant interaction between wheel and diet [*F*_(1, 32)_ = 4.39, *p* = 0.04] on body weight gain. Mice with locked wheels consuming HFD gained more weight than chow-fed mice (^*^p < 0.001). There were significant main effects of wheel [*F*_(1, 32)_ = 27.15, *p* < 0.001] and diet [*F*_(1, 32)_ = 8.28, *p* < 0.01] on food intake. Locked/chow *n* = 7; Locked/HFD *n* = 12; Free/chow *n* = 6; Free/HFD *n* = 11.

We first measured weight gain (Figure 1A) and food intake (Figure 1B) in male mice that were single-housed with either a locked wheel (the wheel was present but could not rotate) or a freely rotating wheel. Body weight did not differ between the groups at baseline when the mice were singly housed (7 weeks old) nor after 1 week of single-housing (8 weeks old; Table S1). All mice were fed chow for 1 week; mice with running wheels consumed significantly more chow (in kcal of metabolizable energy) than mice with locked wheels [*F*_(1, 32)_ = 10.53, *p* < 0.01, Table S1]. Mice were then fed either chow or HFD (45% kcal fat) for the subsequent week. Mice with locked wheels consuming HFD gained more weight than chow-fed mice [*F*_(1, 32)_ = 4.39, *p* = 0.04, *post-hoc p* < 0.001 Figure 1A]. In contrast, when mice were allowed to run on the wheels, weight gain did not differ between mice fed chow and HFD (Figure 1A). Food intake was significantly increased by both wheel-running activity and HFD [Figure 1B; main effect of wheel: *F*_(1, 32)_ = 27.15, *p* < 0.001; main effect of diet: *F*_(1, 32)_ = 8.28, *p* < 0.01].

### High-fat diet and wheel-running activity independently alter circadian organization

**Figure 2:**
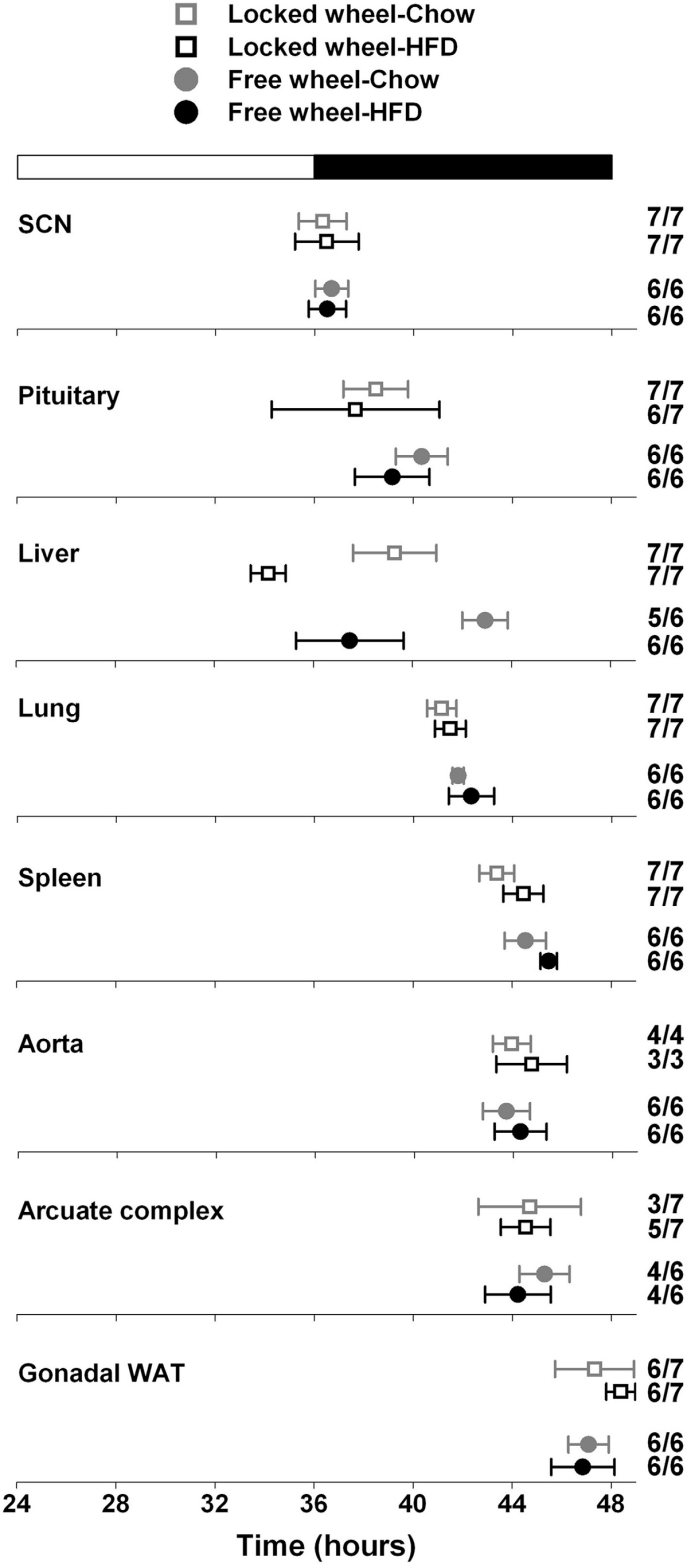
**Wheel-running activity affects the phase of the liver clock**. Male heterozygous PER2::LUC mice were single-housed in cages with either locked (open squares) or freely rotating (filled circles) running wheels. Mice were fed chow (gray symbols) or HFD (black symbols) for 1 week. The mean (±*SD*) phases were determined from the peaks of PER2::LUC expression during the interval between 12 and 36 h in culture and were plotted relative to the time of last lights on where 24 h is lights on and 36 h is lights off (white and black bar at top). The sample size is shown (number of rhythmic tissues/number of tissues tested). Locked wheel data is taken from Pendergast et al. (2013). There were main effects of wheel on the phases of the lung [*F*_(1, 22)_ = 9.22, *p* = 0.006], liver [*F*_(1, 20)_ = 30.72, *p* < 0.001], and spleen [*F*_(1, 21)_ = 15.29, *p* < 0.001] and main effects of diet on the phases of the liver (*F* = 71.25, *p* < 0.001) and spleen (*F* = 13.14, *p* = 0.002). There was no effect of an interaction between wheel and diet on the phase of any tissues.

We next determined the effect of wheel-running activity on the circadian organization of mice fed chow or HFD by measuring the phases of the PER2::LUC bioluminescence rhythms in tissue explants (Figure 2; Table 1). We found that the phases of the aorta, arcuate complex, pituitary, white adipose tissue, and SCN were not altered by wheel-running activity or diet. The phase of the lung was delayed by wheel-running activity in both chow- and HFD-fed mice [main effect of wheel: *F*_(1, 22)_ = 9.22, *p* = 0.006], but was not altered by diet. The phase of the liver was delayed by wheel-running activity [main effect of wheel: *F*_(1, 20)_ = 30.72, *p* < 0.001] and advanced by high-fat diet [main effect of diet: *F*_(1, 20)_ = 71.25, *p* < 0.001], but there was no interaction between wheel activity and diet. Likewise, the phase of the spleen was delayed by wheel-running activity [main effect of wheel: *F*_(1, 21)_ = 15.29, *p* < 0.001] and advanced by HFD [main effect of diet: *F*_(1, 21)_ = 13.14, *p* = 0.002]. Together, these data demonstrate that circadian organization was altered by wheel-running activity or consumption of high-fat diet.

Compared to mice with locked wheels, the periods of the PER2::LUC rhythms in the liver, aorta, and arcuate complex were shortened by wheel-running activity, while the periods of the other tissues were not affected by activity or diet (Table 1). Wheel-running activity increased the amplitude of the PER2::LUC rhythm in the SCN in both chow- and HFD-fed mice [main effect of wheel: *F*_(1, 22)_ = 5.77, *p* = 0.03; Table 1]. The amplitude of the lung was greater in chow-fed compared to HFD-fed mice [main effect of diet: *F*_(1, 22)_ = 5.21, *p* = 0.03; Table 1]. The amplitudes of the other tissues were not altered by activity or diet (Table 1).

### Circadian organization is not altered by environmental enrichment without a running wheel

**Figure 3:**
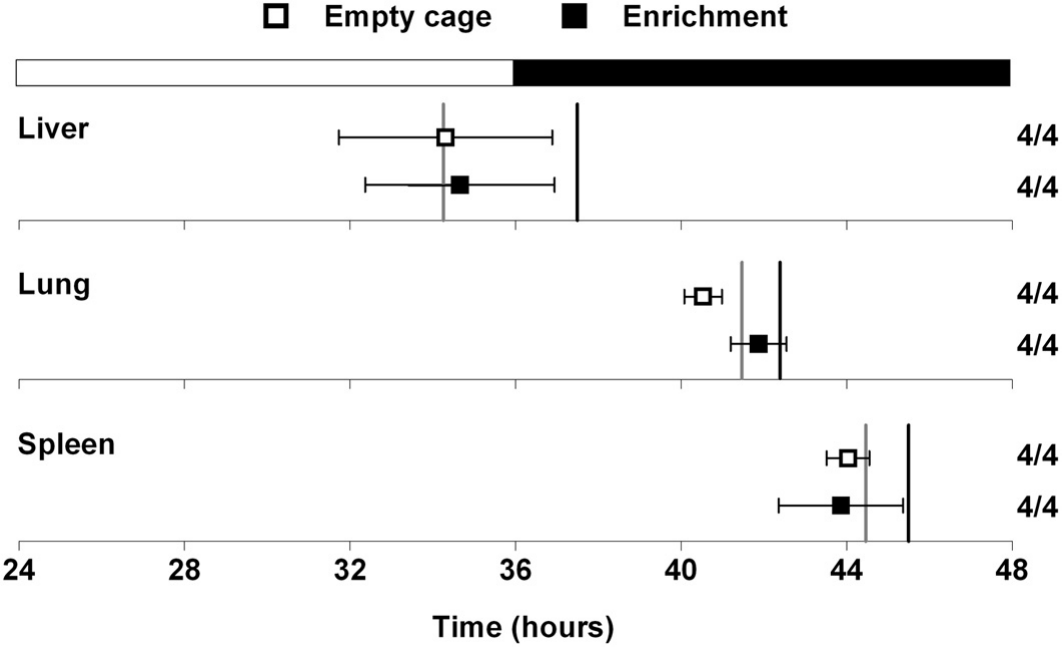
**Effect of environmental enrichment on tissue rhythms**. Male heterozygous PER2::LUC mice were single-housed in either empty cages (open squares) or in cages with enrichment (1 cardboard hut and 2 pieces of shredding paper; filled squares). Mice were fed high-fat diet for 1 week. The mean (±*SD*) phases were determined from the peaks of PER2::LUC expression during the interval between 12 and 36 h in culture and were plotted relative to the time of last lights on where 24 h is lights on and 36 h is lights off (black and white bar at top). The sample size is shown (number of rhythmic tissues/number of tissues tested). The phases of the PER2::LUC rhythms in liver, lung, and spleen were not altered by environmental enrichment. For reference the gray vertical lines show the phases of the tissues from mice housed with locked wheels and the black vertical lines show the phases of the tissues from mice with freely rotating wheels (data shown in Figure 2).

A previous study demonstrated that mice in an enriched environment which included running wheels, toys, and mazes were protected from diet-induced obesity (Cao et al., 2011). Since we found that wheel-running activity delayed the phase of the liver (Figure 2), we next examined whether this effect was specific to wheel-running activity, or whether environmental enrichment without a running wheel could alter circadian organization. Mice were single-housed in either empty cages or in cages with environmental enrichment (1 refuge hut and 2 nesting papers) and fed HFD for 1 week (Figure 3). HFD consumption for 1 week caused body weight gain in mice housed in both the enriched environment (2.23 ± 0.51 g) and in empty cages (1.65 ± 0.19 g). There were no differences in body weight gain or food intake between mice housed in empty cages or in cages with environmental enrichment (Table S2). The phases of the PER2::LUC rhythms in liver, lung, and spleen were not altered by environmental enrichment (Figure 3). These data demonstrate that alteration of liver phase in mice housed with running wheels is specific to wheel-running activity and not attributed to an enriched environment.

### Effects of diet and wheel-running activity on locomotor activity rhythms

**Figure 4:**
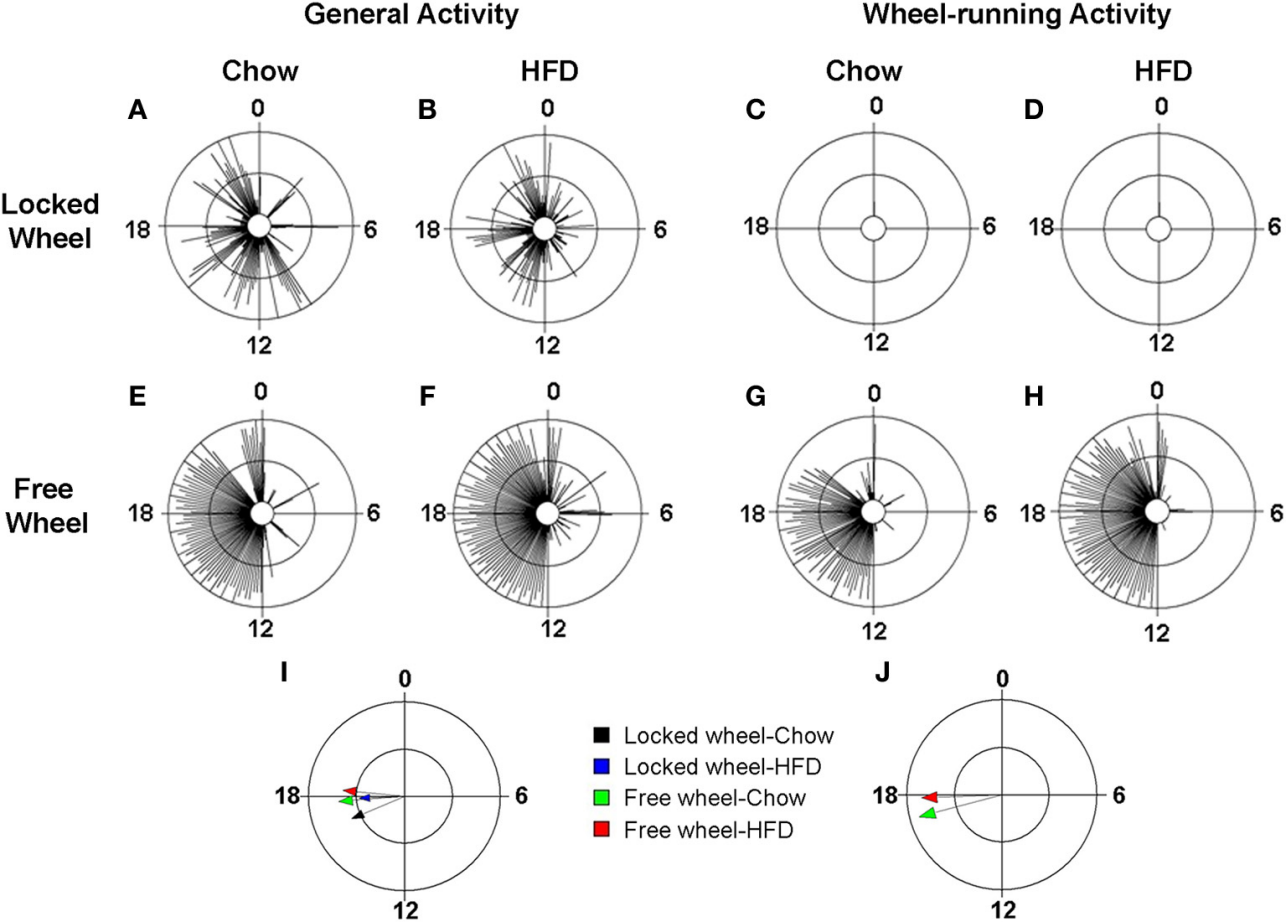
**Locomotor activity rhythms in chow and high-fat diet-fed mice**. Male wild-type mice were single-housed in 12L: 12D with locked **(A–D)** or freely rotating **(E–H)** running wheels at 7 weeks old. Chow was provided *ad libitum* for 1 week (Days 1–7) and then chow was replaced with high-fat diet (HFD) for 1 week (Days 8–15). Representative circular histograms (**A–H**; plotted in 10-min bins; scale: inner circle, 0; middle circle, 5.5, outer circle, 11; units: activity counts per 10-min bin) show the distribution of general activity (**A,B,E,F**; measured with a passive infrared sensor) and wheel activity (**G,H**; no wheel revs in **C,D** because wheel was locked) for a mouse during chow (Day 7; left panels) and HFD (Day 9; right panels) consumption relative to the time of day (where 0 is lights on and 12 is lights off). Grand mean vectors of general activity (**I**; *n* = 5 mice/group) and wheel-running activity (**J**; *n* = 5 mice/group) during chow (black and green arrows) or HFD (blue and red arrows) feeding in mice with locked (black and blue arrows) or freely-rotating wheels are plotted on the circular histogram (plotted in 10-min bins; scale: inner circle, 0.35; outer circle, 0.7). The size and length of the arrow represents the uniformity of the distribution of activity where small, short arrows indicate that activity is more evenly distributed across the cycle.

We next determined the effect of diet and wheel-running on daily rhythms of locomotor activity (Figure 4; Figures S3, S4). To visualize the distribution of activity over the 24-h day, we plotted locomotor activity in circular histograms. The phase and distribution of general activity (Figures 4A,B,E,F) and wheel-running activity (Figures 4G,H) were quantified for each mouse by determining the direction and length, respectively, of the vector of daily activity (Tables S3, S4). Consistent with our previous study, the pattern of general activity in mice with locked wheels, measured by passive infrared sensors, changed immediately upon addition of HFD (Figure S1A; Figures 4A,B). During chow consumption, mice had 5 to 6 bouts of consolidated activity during the light phase and these bouts dissipated during HFD consumption. A similar HFD-induced change in the pattern of general activity was observed in mice with freely rotating wheels (Figure S1C; Figures 4E,F). Compared to chow feeding, the mean phase of the general activity rhythm was delayed by HFD consumption in mice with either locked or freely rotating wheels (Figure 4I). Similarly, the phase of the wheel-running activity rhythm was delayed by HFD consumption compared to chow (Figure 4J).

### Wheel-running activity modulates the effect of high-fat diet consumption on the eating behavior rhythm

**Figure 5:**
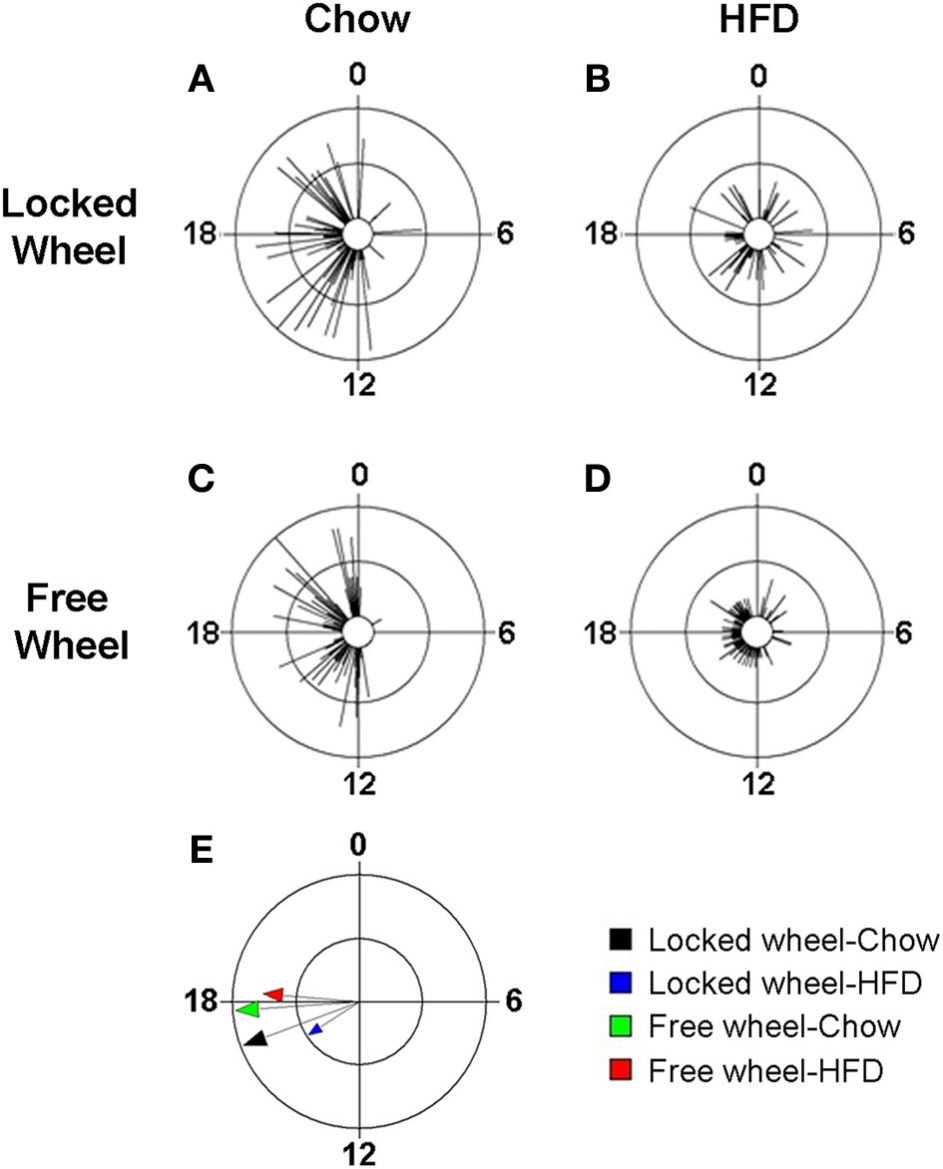
**Wheel-running activity alters the eating behavior rhythm in mice fed high-fat diet**. Male wild-type mice were single-housed in 12L: 12D with a locked **(A,B)** or freely rotating **(C,D)** running wheels at 7 weeks old. Chow was provided *ad libitum* for 1 week (Days 1–7) and then chow was replaced with high-fat diet (HFD) for 1 week (Days 8–15). Representative circular histograms (**A–D**; plotted in 10-min bins; scale: inner circle, 0; middle circle, 5.5, outer circle, 11; units: eating events per 10-min bin) show the distribution of eating events for a mouse during chow (Day 7; left panels) and HFD (Day 9; right panels) consumption relative to the time of day (where 0 is lights on and 12 is lights off). Grand mean vectors of eating behavior (**E**; *n* = 5 mice/group) during chow (black and green arrows) or HFD (blue and red arrows) feeding in mice with locked (black and blue arrows) or freely-rotating wheels are plotted on the circular histogram (plotted in 10-min bins; scale: inner circle, 0.3; outer circle, 0.6). The size and length of the arrow represents the uniformity of the distribution of the eating events where small, short arrows indicate that eating events are more evenly distributed across the cycle.

We previously found that high-fat diet altered the daily rhythm of eating behavior (Pendergast et al., 2013). To determine if wheel-running activity could modify the effect of HFD consumption on the daily rhythm of eating behavior, we continuously monitored eating events with an infrared camera and plotted eating events in circular histograms (Figures 5, S5). The phase and distribution of eating events were quantified for each mouse by determining the direction and length, respectively, of the vector of daily eating behavior (Figures 5A–D; Table S5). When mice were provided with chow, eating events were consolidated during the night (Figures 5A,C) and the direction and length of the mean vectors of eating behavior did not differ between mice with locked or freely rotating wheels (Figure 5E: black and green vectors; Table S5). Consistent with our previous results, the eating behavior rhythm was rapidly altered (within 1 day) in mice given HFD with locked wheels such that eating events became more evenly distributed across the day and night (Figure 5B), as indicated by a decrease in the length of the mean vector of eating behavior (Figure 5E: blue vector; Table S5). However, when mice had freely rotating wheels, the effect of HFD consumption on the distribution of eating events was less marked (Figure 5D). With running wheel activity, eating events occurred mostly during the night, with some events occurring during the day (Figure 5D). The length of the mean vector of HFD eating events for wheel-running mice (Figure 5E: red vector; Table S4) was intermediate to that of HFD eating in mice with locked wheels (Figure 5E: blue vector) and mice consuming chow (Figure 5E: black and blue vectors). The mean phase of the eating events was delayed in wheel-running mice (Figure 5E: green and red vectors; Table S4) compared to mice with locked wheels (Figure 5E: black and blue vectors).

## Discussion

Voluntary wheel-running activity prevents the obesogenic effects of high-fat diet in mice (Krawczewski Carhuatanta et al., 2011). We previously showed that after one week of HFD consumption, mice experienced a 10% increase in body mass, the phase of their liver clock was advanced by 5 h, and their eating behavior rhythm was less robust (Pendergast et al., 2013). Since wheel-running activity is effective in preventing diet-induced obesity and also has potent effects on circadian parameters of behavior, we postulated that it would modulate the effects of HFD consumption on tissue and behavior rhythms.

In our previous study, we identified the liver clock as a proximate target of high-fat diet consumption (Pendergast et al., 2013). After only 1 week of high-fat diet feeding in mice with locked wheels, the phase of the PER2::LUC rhythm in the liver was advanced by 5 h compared to chow-fed controls. In this study, we found that wheel-running activity delayed the phase of the liver PER2::LUC rhythm by 3 h compared to mice housed with locked wheels in both chow- and HFD-fed mice. Thus, the magnitude of the phase advance of the liver rhythm caused by HFD consumption was equivalent in wheel-running and locked-wheel mice. These data demonstrate that both wheel-running activity and HFD can alter the phase of the liver.

A complex enriched environment consisting of group-housed mice in cages with running wheels, mazes, and toys prevented weight gain in mice fed HFD for 4 weeks (Cao et al., 2011). In mice, consumption of HFD is akin to administration of drugs of abuse and environmental enrichment blunts the rewarding effects of drugs (Xu et al., 2007; El Rawas et al., 2009; Pandit et al., 2012). Thus it is possible that environmental enrichment decreases the reward associated with HFD, resulting in protection from diet-induced obesity. However, enriched environments typically contain a running wheel, so it is difficult to distinguish the effects of running wheel activity, which increases exercise and is itself a unique motivated behavior, from the other enhanced sensory and cognitive experiences of the enriched environment. We determined if the delay of the liver phase was specific to wheel-running activity or if an enriched environment without the running wheel could alter circadian organization. We found that circadian organization did not differ between HFD-fed mice in empty cages and those in cages with environmental enrichment without a running wheel. These findings suggest that the running wheel specifically alters circadian organization.

We found that the periods of the PER2::LUC rhythms in liver, aorta, and arcuate nucleus of the hypothalamus were shorter in wheel-running compared to locked-wheel mice. We do not know how wheel-running activity results in the shortening of the periods of tissue rhythms. However, it has been demonstrated that wheel-running activity alters physiological processes and gene expression in liver, aorta, and hypothalamus and it is possible that these changes could relate to alterations in the period of the circadian rhythm (Haskell-Luevano et al., 2009; Werner et al., 2009; Kim et al., 2010).

In this study, we examined the effect of HFD and wheel-running activity on three different behavioral outputs. We first assessed general activity measured by passive infrared sensors, which detected the spectrum of mouse behaviors, including grooming, eating, drinking, nesting, and exploring (climbing, sniffing, digging). We found that regardless of whether the mice had locked or freely rotating wheels, HFD consumption had similar effects on the pattern of general activity such that consolidated daytime bouts of activity dissipated during HFD. HFD consumption delayed the phase of general activity in both locked and wheel-running mice, but the distribution of activity (length of vector) was affected only in mice with locked wheels. General activity became more evenly distributed across the day and night during HFD consumption by mice with locked wheels.

We also measured wheel-running activity, which is postulated to be a motivated and/or rewarding behavior in mice (Sherwin, 1998). We found that the phase of the wheel-running rhythm was delayed by consumption of HFD compared to chow, but the number of wheel revolutions and the distribution of wheel activity (length of vector) were not altered by HFD.

We also specifically measured eating behavior by continuously recording mouse behavior at the feeder and scoring eating events. In contrast to general activity, we found that wheel-running activity modulated the effect of HFD consumption on the daily rhythm of eating behavior. While HFD consumption caused eating events to be more evenly dispersed across the 24 h-day in both locked-wheel and wheel-running mice, the effect of HFD was much less pronounced in wheel-running mice.

For both eating behavior and liver rhythms, wheel-running activity caused phase delays but the magnitudes of the delays in behavior and in the phases of liver explants were different (Figure S6). Thus, we hypothesize that HFD consumption and wheel-running activity act through distinct pathways to alter liver phase, but they converge to modulate eating behavior. These data suggest that the phase of the liver cannot be solely explained by the change in the eating behavior rhythm during *ad libitum* feeding. This is in contrast to previous studies where restricted meal timing entrained the phase of the liver (Stokkan et al., 2001, but see Hatori et al., 2012 for restricted feeding of high-fat diet). There are a couple of potential explanations for the apparent disconnect between the eating behavior rhythm and liver phase. First, we analyzed the eating behavior rhythm on the first full day of high-fat consumption while liver phase was analyzed after 1 week of HFD consumption. Second, future studies should examine whether the phases of food intake rhythms (as opposed to eating behavior) and liver rhythms are correlated in mice with locked and freely rotating wheels.

Wheel-running activity in mice is both a source of exercise and a self-motivating, rewarding behavior. It is unclear whether it is the exercise, the reward, or both properties of wheel-running activity that alter liver phase and the daily rhythm of eating behavior. Previous studies have demonstrated that wheel-running activity is salient in modulating circadian rhythms in rodents, while forced treadmill exercise may not be as effective, thus it has been hypothesized that wheel-running activity may be a unique motivational state that impacts upon circadian rhythmicity (Mistlberger, 1991; Janik and Mrosovsky, 1993; Mistlberger et al., 1996 but see Marchant and Mistlberger, 1996; Wolff and Esser, 2012). Future experiments could examine the effect of forced treadmill running, which provides exercise without self-motivated reward, on circadian organization and eating behavior. Understanding the putative reward-related mechanisms whereby wheel-running activity alters circadian rhythms could have implications for human obesity since palatable food (like drugs of addiction) is rewarding and may promote dependence (Volkow et al., 2012).

## Author contributions

Julie S. Pendergast designed and performed experiments, analyzed data, and wrote the manuscript. Katrina L. Branecky and Roya Huang performed experiments and analyzed data. Kevin D. Niswender contributed to the design of the experiments and participated in discussion and writing of the manuscript. Shin Yamazaki designed and performed experiments and wrote the manuscript.

### Conflict of interest statement

The authors declare that the research was conducted in the absence of any commercial or financial relationships that could be construed as a potential conflict of interest.
