# Supplementary material for: Wheel-running activity modulates circadian organization and the daily rhythm of eating behavior
Source: Front Psychol. 2014 Mar 4;5:177. doi: 10.3389/fpsyg.2014.00177 (PMC3941004; doi:10.3389/fpsyg.2014.00177)
Supplement: Supplementary file 1 [file DataSheet1.PDF]

Supplemental Information

Table S1. Body weight and food intake in experimental groups

		**Locked Wheel**		**Free Wheel**		
	**Age**	**Chow: mean±SD (n)**	**HFD: mean±SD (n)**	**Chow: mean±SD (n)**	**HFD: mean±SD (n)**	***P****
**Body weight (g)**	7 weeks	20.67±1.37 (7)	21.12±1.54 (12)	20.10±2.46 (6)	20.91±1.65 (11)	NS
	8 weeks	21.43±1.44 (7)	21.33±1.48 (12)	20.77±2.15 (6)	21.89±1.54 (11)	NS
	9 weeks	21.46±1.47 (7)	23.12±1.82 (12)	21.50±2.06 (6)	23.15±1.59 (11)	Diet; $F_{1,32}$=7.53, *p*=0.01
**Food intake (kcal)**	7-8 weeks	90.04±5.76 (7)	80.79±6.41 (12)	92.21±10.75 (6)	96.28±8.56 (11)	Wheel: $F_{1,32}$=10.53, p<0.01
	8-9 weeks	84.52±10.62 (7)	94.21±12.28 (12)	104.49±14.23 (6)	120.191±13.40 (11)	Wheel: $F_{1,32}$=27.15, *p*<0.001; Diet: $F_{1,32}$=8.28, *p*<0.01

*The data were compared by 2 x 2 factorial analysis. None of the tissues had a significant interaction between wheel and diet. Some tissues showed a significant effect of wheel or diet, as reported in the table. NS indicates that no significant differences were found.

Table S2. Body weight and food intake in environmental enrichment experimental groups

	**Age**	**Empty cage: mean±SD (n=4)**	**Enrichment: mean±SD (n=4)**	***P****
**Body weight (g)**	7 weeks	22.43±1.95	22.6±3.36	NS
	8 weeks	22.48±1.99	22.90±2.84	NS
	9 weeks	24.13±2.11	25.13±3.05	NS
**Food intake (kcal)**	7-8 weeks (chow)	91.40±7.46	102.00±21.10	NS
	8-9 weeks (HFD)	101.00±8.51	116.00±10.90	NS

*All mice were fed chow during from age 7-8 weeks and high-fat diet (HFD) from age 8-9 weeks. The data were compared by independent t-tests (two-tailed). NS indicates that no significant differences were found.

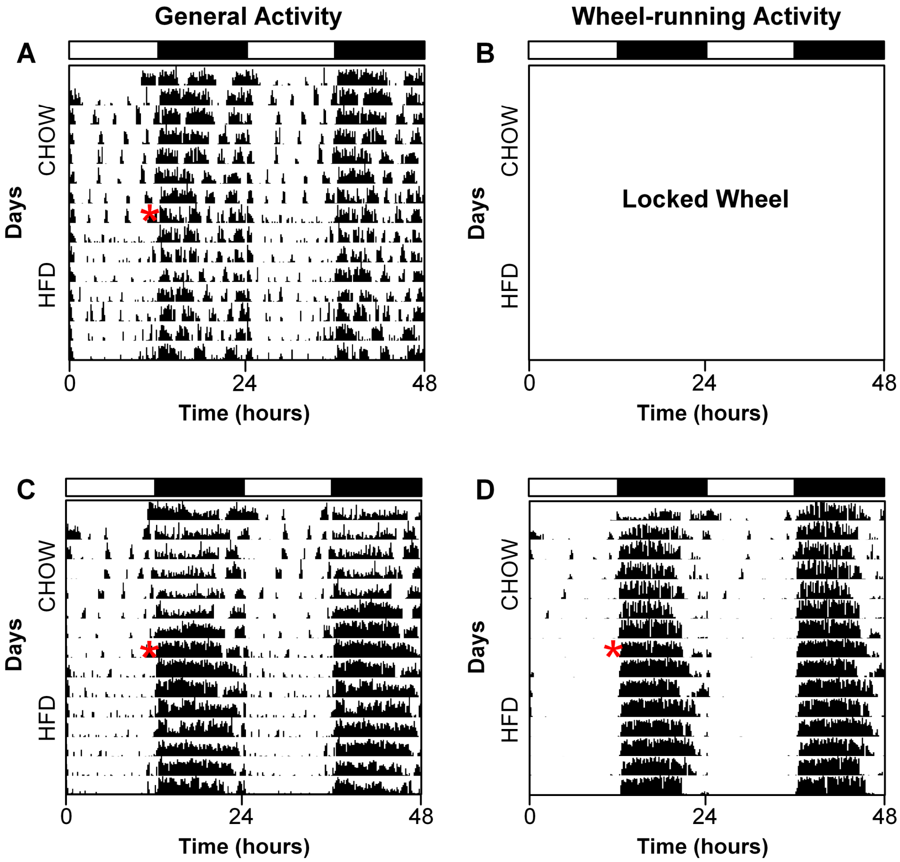

Figure S1. Locomotor activity in chow- and high-fat diet-fed mice. Representative double-plotted actograms (10-min bins) of male heterozygous PER2::LUC mice maintained in 12L:12D (light and dark indicated by black and white bars, respectively, above actograms). One mouse (A, B) was housed with a locked wheel and another mouse (C, D) was housed with a freely rotating wheel. General activity was continuously monitored with passive infrared sensors. All mice were provided with chow ad libitum for the first week. After 1 week, chow was replaced with high-fat diet (HFD). The times when food was replaced are indicated by red asterisks on the left halves of the actograms. The mouse shown in A, B is also shown in Fig. 3A-D (mouse #6335). The mouse shown in C,D is also shown in Fig. 3E-H (mouse #6450).

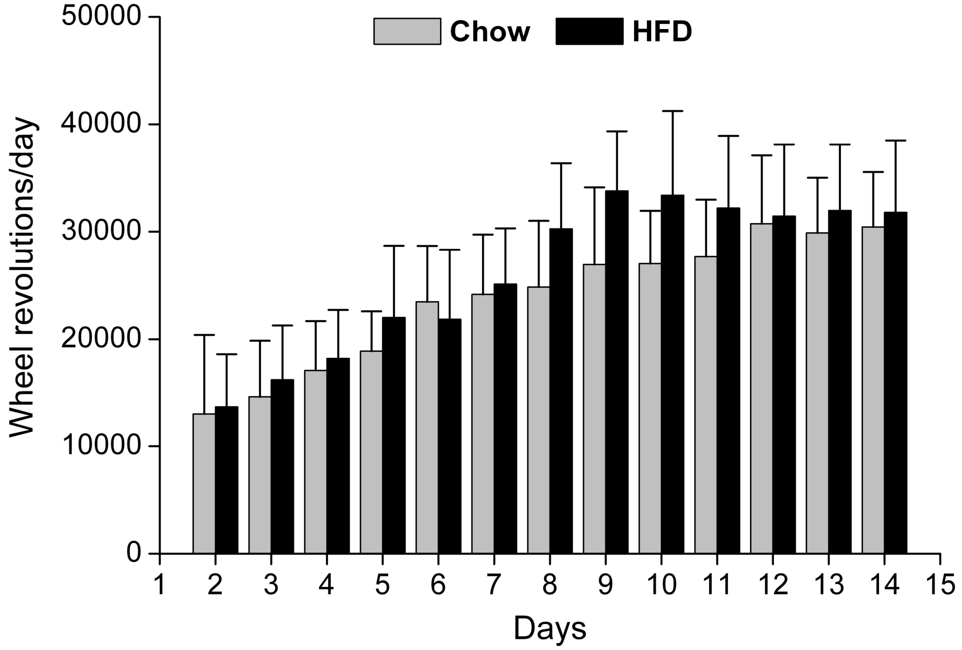

Figure S2. Wheel revolutions per day in chow- and high-fat diet-fed mice. Male wild-type mice were single-housed in 12L:12D with freely rotating running wheels at 7 weeks old. Chow was provided *ad libitum* for 1 week (days 1-7). On day 8, chow was replaced with either fresh chow (gray; n=6) or with HFD (black; n=11) for 1 week (days 8-15). The mean number of wheel revolutions per 24h-day ±SD are shown.

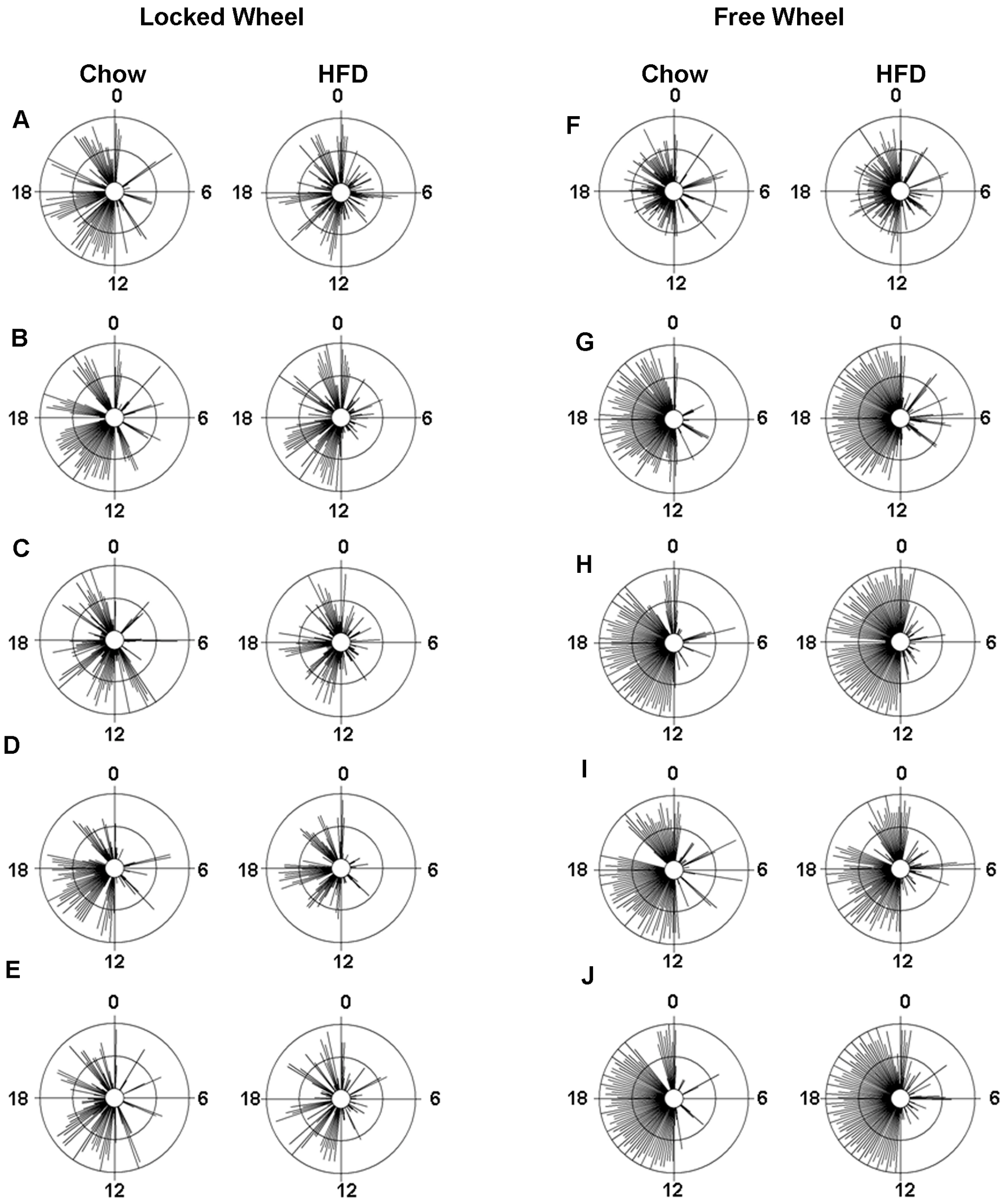

Figure S3. Distribution of general activity in individual mice during consumption of chow and HFD. Male wild-type mice maintained in 12L:12D were single housed with a locked (A-E) or freely rotating (F-J) running wheel at 7 weeks old. Chow was provided *ad libitum* for 1 week (Days 1-7) and then chow was replaced with HFD for 1 week (Days 8-15). Circular histograms (plotted in 2.5° bins; scale: inner circle, 0; middle circle, 5.5, outer circle, 11) show the distribution of activity for each mouse during chow (Day 7; left panels) and HFD (Day 9; right panels) consumption relative to the time of day (where ZT0 is lights on and ZT12 is lights off). The mean vectors for each mouse are reported in Table 2. Data from all individual mice are presented (A: mouse #6334; B: mouse #6335; C: mouse #6339 (this data also shown in Fig. 3); D: mouse #6305; E: mouse #6456; F: mouse #6385; G: mouse #6341; H: mouse #6328; I: mouse #6476; J: mouse #6450 (this data also shown in Fig. 3).

Table S3. Vector properties of rhythms of general activity in individual mice.

		**Chow**			**High-fat diet**		
**Wheel?**	**Mouse ID**	**Mean angle (µ)±SD**	**Length (r)**	***P****	**Mean angle (µ)±SD**	**Length (r)**	***P****
Locked	#6334	256±76	0.41	Z=83.3, *p*< 1E-12	275±100	0.22	Z=19.0, *p*= 5.8E-09
	#6335	248±75	0.42	Z=92.6, *p*< 1E-12	265±82	0.36	Z=59.5, *p*< 1E-12
	#6339	236±85	0.33	Z=48.9, *p*< 1E-12	270±86	0.33	Z=39.0, *p*< 1E-12
	#6305	249±65	0.53	Z=119.1, *p*< 1E-12	270±66	0.52	Z=80.2, *p*< 1E-12
	#6456	242±75	0.43	Z=74.8, *p*< 1E-12	260±85	0.33	Z=36.3, *p*< 1E-12
	Grand mean vector†	247 (227-260)	0.42	F=80.3, *p*=0.002	268 (252-282)	0.35	F=19.5, *p*=0.02
Free	#6385	283±89	0.30	Z=34.8, *p*<1E-12	281±81	0.37	Z=58.4, *p*<1E-12
	#6341	270±61	0.57	Z=204.2, *p*<1E-12	281±69	0.49	Z=176.4, *p*<1E-12
	#6328	260±61	0.56	Z=211.0, *p*< 1E-12	275±69	0.49	Z=182.7, *p*< 1E-12
	#6476	261±76	0.42	Z=113.3, *p*< 1E-12	267±78	0.40	Z=95.6, *p*< 1E-12
	#6450	260±59	0.60	Z=231.7, *p*< 1E-12	273±67	0.51	Z=197.0, *p*< 1E-12
	Grand mean vector†	265 (252-297)	0.48	F=41.6, p=0.007	276 (262-288)	0.45	F=93.1, p=0.002

The mean angle (µ) ± circular standard deviation (SD) and vector length (r) are reported are for individual mice. *Rayleigh's Uniformity test was used to determine if the eating events of individual mice had a significant non-uniform direction (for individual mice). †Hotelling's one sample test was used to test if there was a significant mean direction (for grand mean vectors). The 95% confidence intervals are reported (in parentheses) for the directions of the grand mean vectors.

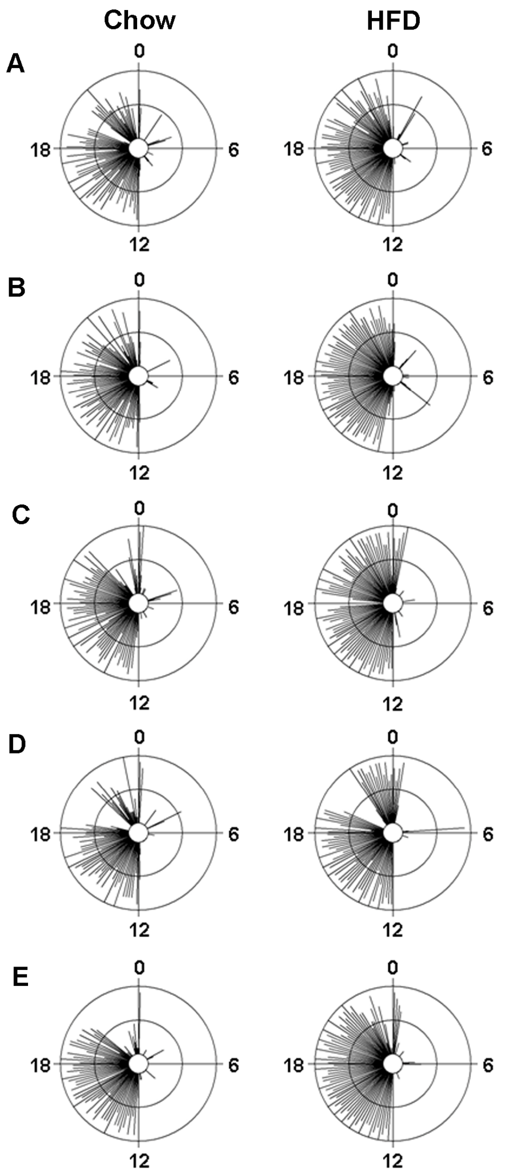

Figure S4. Distribution of wheel running activity events in individual mice during consumption of chow and HFD. Male wild-type mice maintained in 12L:12D were single housed with a freely rotating running wheel at 7 weeks old. Chow was provided ad libitum for 1 week (Days 1-7) and then chow was replaced with HFD for 1 week (Days 8-15). Circular histograms (plotted in 2.5° bins; scale: inner circle, 0; middle circle, 5.5, outer circle, 9) show the distribution of wheel-running events for each mouse during chow (Day 7 with running wheel, left panels) and HFD (Day 9 with running wheel; right panels) consumption relative to the time of day (where ZT0 is lights on and ZT12 is lights off). The mean vectors for each mouse are reported in Table 3. Each row of data is taken from a single mouse (A: mouse #6385; B: mouse #6341; C: mouse #6328; D: mouse #6476; E: mouse #6450 (this data also shown in Fig. 3). Eating behavior was simultaneously recorded in these mice (eating behavior histograms presented in Figure S5).

Table S4. Vector properties of wheel-running activity rhythms in individual mice.

		Chow			High-fat diet		
**Wheel?**	**Mouse ID**	**Mean angle (μ)±SD**	**Length (r)**	***P****	**Mean angle (μ)±SD**	**Length (r)**	***P****
Free	#6385	258±58	0.59	Z=173.7, *p*<1E-12	264±56	0.63	Z=238.4, *p*<1E-12
	#6341	261±55	0.64	Z=207.7, *p*<1E-12	272±56	0.62	Z=257.5, *p*<1E-12
	#6328	260±56	0.63	Z=202.9, *p*< 1E-12	273±62	0.55	Z=208.5, *p*< 1E-12
	#6476	248±59	0.59	Z=155.8, *p*< 1E-12	264±65	0.53	Z=166.9, *p*< 1E-12
	#6450	249±47	0.71	Z=243.3, *p*< 1E-12	266±55	0.63	Z=262.9, *p*< 1E-12
	Grand mean vector†	255 (242-270)	0.63	F=343.6, *p*=2.9E-04	268 (259-278)	0.59	F=281.7, *p*=3.9E-04

The mean angle (μ) ± circular standard deviation (SD) and vector length (r) are reported are for individual mice. *Rayleigh's Uniformity test was used to determine if the eating events of individual mice had a significant non-uniform direction (for individual mice). †Hotelling's one sample test was used to test if there was a significant mean direction (for grand mean vectors). The 95% confidence intervals are reported (in parentheses) for the directions of the grand mean vectors.

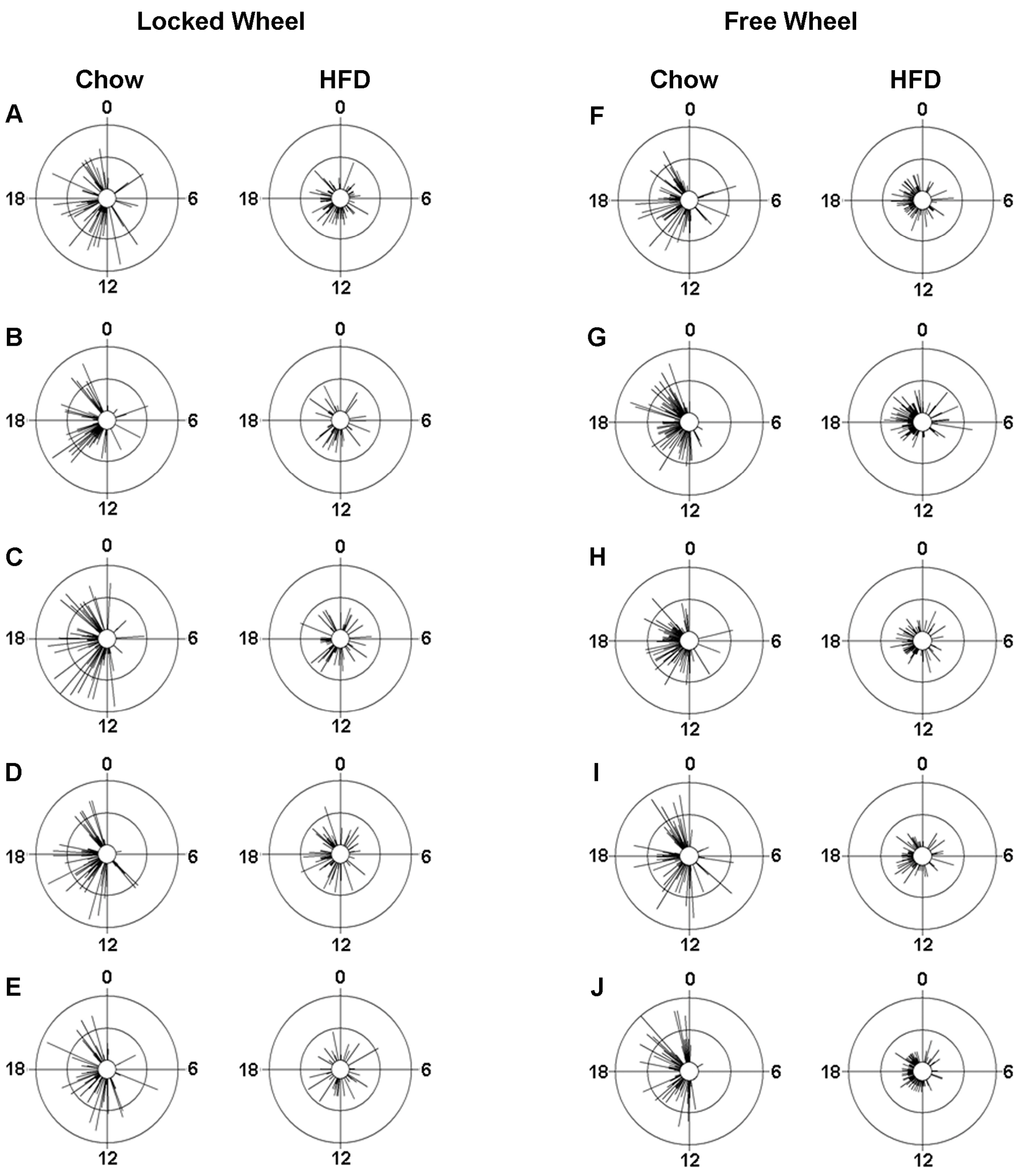

Figure S5. Distribution of eating events in individual mice during consumption of chow and HFD. Male wild-type mice maintained in 12L:12D were single housed with a locked (A-E) or freely rotating (F-J) running wheel at 7 weeks old. Chow was provided *ad libitum* for 1 week (Days 1-7) and then chow was replaced with HFD for 1 week (Days 8-15). Circular histograms (plotted in 2.5° bins; scale: inner circle, 0; middle circle, 5.5, outer circle, 11) show the distribution of eating events for each mouse during chow (Day 7; left panels) and HFD (Day 9; right panels) consumption relative to the time of day (where 0 is lights on and 12 is lights off). The mean vectors for each mouse are reported in Table 4. Data from all individual mice are presented (A: mouse #6334; B: mouse #6335; C: mouse #6339 (this data also shown in Fig. 4); D: mouse #6305; E: mouse #6456; F: mouse #6385; G: mouse #6341; H: mouse #6328; I: mouse #6476; J: mouse #6450 (this data also shown in Fig. 4).

Table S5. Vector properties of eating behavior rhythms in individual mice.

		Chow			High-fat diet		
**Wheel?**	**Mouse ID**	**Mean angle(μ) ± SD**	**Length (r)**	***P****	**Mean angle (μ) ± SD**	**Length (r)**	***P****
Locked	#6334	241±76	0.41	Z=35.2, *p*< 1E-12	233±86	0.32	Z=11.2, *p*< 1.4E-05
	#6335	256±62	0.55	Z=55.4, *p*< 1E-12	211±91	0.28	Z=6.5, *p*=.001
	#6339	256±66	0.52	Z=65.3, *p*< 1E-12	249±103	0.20	Z=4.3, *p*=.01
	#6305	250±63	0.55	Z=64.0, *p*< 1E-12	270±85	0.34	Z=14.4, *p*= 5.8E-07
	#6456	241±76	0.41	Z=30.6, *p*< 1E-12	202±112	0.15	Z=1.8, *p*= .17
	Grand mean vector†	250 (227-262)	0.49	F=350.5, *p*= 2.8E-04	237 (168-286)	0.24	F=19.9, *p*= .02
Free	#6385	257±69	0.48	Z=44.1, *p*<1E-12	274±76	0.42	Z=19.4, *p*=3.0E-09
	#6341	268±59	0.59	Z=80.3, *p*<1E-12	295±78	0.39	Z=25.4, *p*=9.7E-12
	#6328	257±62	0.56	Z=60.2, *p*< 1E-12	262±83	0.35	Z=11.7, *p*= 8.7E-06
	#6476	268±80	0.37	Z=30.1, *p*< 1E-12	267±81	0.36	Z=12.6, *p*= 3.4E-06
	#6450	281±67	0.50	Z=49.3, *p*< 1E-12	274±76	0.41	Z=17.8, *p*= 2.0E-08
	Grand mean vector†	266.0 (240-290)	0.49	F=65.4, *p*=0.003	275 (247-301)	0.39	F=232.8, *p*= 5.1E-04

The mean angle (μ) ± circular standard deviation (SD) and vector length (r) are reported are for individual mice. *Rayleigh's Uniformity test was used to determine if the eating events of individual mice had a significant non-uniform direction (for individual mice). †Hotelling's one sample test was used to test if there was a significant mean direction (for grand mean vectors). The 95% confidence intervals are reported (in parentheses) for the directions of the grand mean vectors.

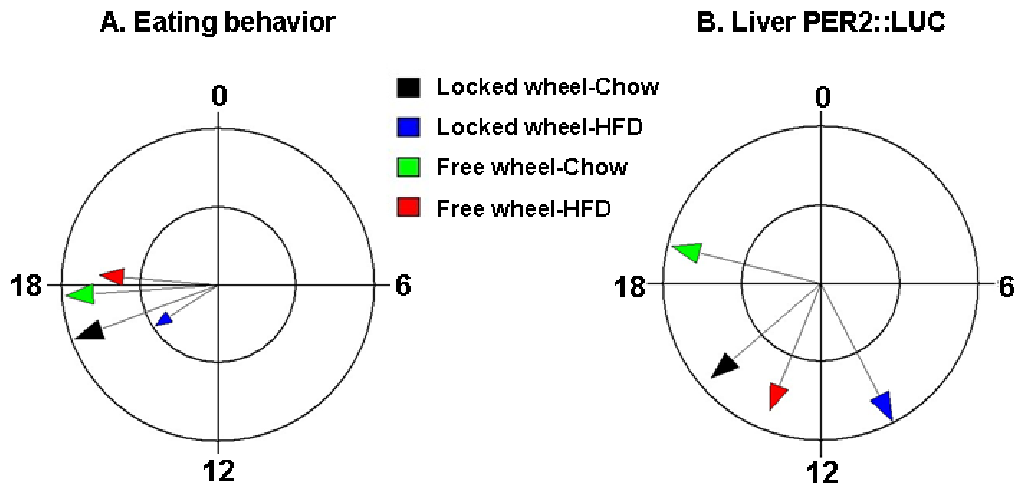

Figure S6. Mean vectors of eating behavior and liver rhythms. Grand mean vectors of eating behavior (n=5 mice/group) and liver PER2::LUC phase (n=5-7/group as indicated in Figure 2). Circular histograms (plotted in 2.5° bins; scale: inner circle, .3; outer circle, .6) show the distribution of eating events for each mouse during chow (Day 7; left panels) and HFD (Day 9; right panels) consumption relative to the time of day (where 0 is lights on and 12 is lights off).
